# Evaluation of quality improvement for cesarean sections programmes through mixed methods

**DOI:** 10.1186/s13012-014-0182-0

**Published:** 2014-12-11

**Authors:** Clara Bermúdez-Tamayo, Mira Johri, Francisco Jose Perez-Ramos, Gracia Maroto-Navarro, Africa Caño-Aguilar, Leticia Garcia-Mochon, Longinos Aceituno, François Audibert, Nils Chaillet

**Affiliations:** Centre de recherche du CHUS, 12e Avenue Nord, Sherbrooke, QC J1H 5 N4 Canada; Andalusian School of Public Health, Cuesta del Observatorio 4 s/n, 18010 Granada, Spain; CIBERESP, Ciber de Epidemiologia y Salud Publica, València, Spain; Division of Global Health, University of Montreal, Hospital Research Centre (CRCHUM), 900, rue Saint-Denis, H2X 0A9 Montreal, QC Canada; Department of Health Administration, School of Public Health, University of Montreal, Montreal, QC Canada; General Secretary of Quality iInnovation and Public Health, Consejería de Igualdad, Salud y Políticas Sociales, Junta de Andalucía, Avd. De Hytasa n° 14, 41006 Sevilla, Spain; UGC Obstetrics and Gynaecology, Hospital Universitario San Cecilio, Av Doctor Oloriz, 16, 18012 Granada, Spain; UGC Gynaecology, Hospital La Inmaculada, Av. Dra. Parra, S/N., 04600 Huercal-Overa, Almeria Spain; Department of Obstetrics and Gynecology, University of Montreal, Montreal, QC Canada; Sainte Justine Hospital, 3175 Chemin de la Côte-Sainte-Catherine, Montreal, QC H3T 1C5 Canada; Department of Obstetrics and Gynaecology, Université de Sherbrooke, 12e Avenue Nord, Sherbrooke, QC J1H 5 N4 Canada

**Keywords:** Caesarean section, Clinical practice guidelines, Economic evaluation

## Abstract

**Background:**

The rate of avoidable caesarean sections (CS) could be reduced through multifaceted strategies focusing on the involvement of health professionals and compliance with clinical practice guidelines (CPGs). Quality improvements for CS (QICS) programmes (QICS) based on this approach, have been implemented in Canada and Spain. Objectives Their objectives are as follows: 1) Toto identify clusters in each setting with similar results in terms of cost-consequences, 2) Toto investigate whether demographic, clinical or context characteristics can distinguish these clusters, and 3) Toto explore the implementation of QICS in the 2 regions, in order to identify factors that have been facilitators in changing practices and reducing the use of obstetric intervention, as well as the challenges faced by hospitals in implementing the recommendations.

**Methods:**

Descriptive study with a quantitative and qualitative approach. 1) Cluster analysis at patient level with data from 16 hospitals in Quebec (Canada) (*n* = 105,348) and 15 hospitals in Andalusia (Spain) (*n* = 64,760). The outcome measures are CS and costs. For the cost, we will consider the intervention, delivery and complications in mother and baby, from the hospital perspective. Cluster analysis will be used to identify participants with similar patterns of CS and costs based, and *t* tests will be used to evaluate if the clusters differed in terms of characteristics: Hospital level (academic status of hospital, level of care, supply and demand factors), patient level (mother age, parity, gestational age, previous CS, previous pathology, presentation of the baby, baby birth weight). 2) Analysis of in-depth interviews with obstetricians and midwives in hospitals where the QICS were implemented, to explore the differences in delivery-related practices, and the importance of the different constructs for positive or negative adherence to CPGs. Dimensions: political/management level, hospital level, health professionals, mothers and their birth partner.

**Discussion:**

This work sets out a new approach for programme evaluation, using different techniques to make it possible to take into account the specific context where the programmes were implemented.

## Background

Caesarean section (CS) rates have increased in recent years. Possible causes for the increase include changes in sociodemographic factors and in clinical practices, as well as changes in the attitudes of health professionals and women towards CS [1].

Although the increase in CS rates seems to convey the feeling of safety among patients and professionals, several studies have shown an association between maternal and perinatal morbidity and CS [2-5]. CS can also generate higher costs than vaginal delivery; it has been estimated that a CS is about 44% more expensive [6].

A systematic review of the effectiveness of strategies for reducing CS has shown that the CS rate can be safely reduced through multifaceted interventions that involve health workers in analysing and modifying their practice, taking into account the clinical practice guidelines (CPGs) [7]. Educational intervention projects with health professionals have recently been implemented in Spain and Canada with the aim of decreasing preventable CS, leading to improved quality and patient safety. The projects hypothesizse that poor adherence to CPGs plays a key role in the rising CS rate.

Health professionals, policy-makers and researchers already know that clinical practice is not always based on scientific evidence, either because such evidence does not exist or because the available evidence is not applied by clinicians [8]. In the case of CS, the different CPGs and indications of CS are quite consistent [2]. CPGs would make it easier to transfer the evidence into clinical practice and are a means of reducing CS rates. The challenge lies in their implementation. However, each clinical setting has its own specific organisational, professional and cultural characteristics that can be barriers or facilitators for the knowledge translation [9].

A quality improvement for CS programme called the QUARISMA project has been developed in Quebec (Canada). The steps taken in this programme include the training of professionals in best practices, implementation and technical audit of CS with feedback. The audit process allows inadequate CS practices to be identified and corrective actions to be defined. Training has been conducted in collaboration with the Canadian Society of Gynaecology and Obstetrics in Canada (SOGC). Meanwhile, in Andalusia (Spain)), a multi-centremulticentre project of compliance of CS to clinical standards using a similar methodology has been developed.

The preliminary results of the projects show a decrease in obstetric interventions. However, there is also a significant variation between hospitals. An analysis still needs to be performed to determine which elements of these interventions have been effective, to know how to apply the knowledge that has been generated and to determine how the models can be adapted to change childbirth practices and system performance.

Canada has the distinction of having an extensive background in the definition of guidelines and evidence-based medicine. The Canadian Society of Obstetricians and Gynaecologists in Canada has developed a number of CPGs related to childbirth [10-13]. Spain has also made progress in the area in recent years. In the specific case of obstetric care, the Spanish Ministry of Health has recently developed its own Clinical Practice Guidelines on Care in Normal Childbirth [14], as well as the Spanish Society of Obstetricians and Gynaecologists [15-18].

The aim of this study is to compare the programmes in the two regions, taking into account the two specific contexts, in order to identify the aspects that have been effective in changing practices and reducing the use of obstetric intervention, as well as the challenges faced by hospitals in implementing the recommendations. The factors considered are those related to the political/management level, hospitals, practitioners and patients. Finally, we will analyse the differences in the results of the Qquality improvement for CS programmes (QICS) in both contexts, factors related to better results.

This study is a secondary analysis of both the previous studies. The proposed methodology makes it possible to study the influence of organisational and contextual factors on the results obtained after introducing an innovation based on 3three components [19,20]: first, the influence of contextual determinants in the degree of implementation of the changes; second, the process of implementing the changes; and third, the interaction between the application context and the effects of the intervention.

## Methods

**Figure 1:**
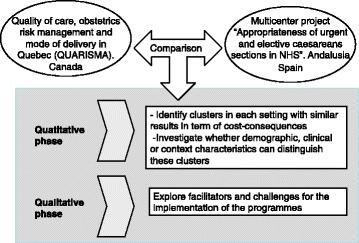
**Study flow diagram.**

Mixed methods and both quantitative and qualitative approaches will be used (Figure 1). The quantitative approach will allow us to identify clusters in each setting with similar results in terms of cost-consequences and to investigate whether demographic, clinical or context characteristics can distinguish these clusters. The qualitative study will allow us to explore and describe the perceptions of obstetricians and midwifves in hospitals in Quebec and Andalusia.

### Setting

The study will be carried out in the hospitals where the QICS have been implemented, in Andalusia and Quebec.

### Cluster analysis

#### Target population

The study population will be mother-infant pairs. The study population will include patients from hospitals in Québec (16 sites) and Andalusia (15 sites) for a total of approximately 170,108 patients, 105,348 from Quebec and 64,760 from Andalusia.

Evidence of effectiveness and costs is based on information collected between 2010–2012 in Quebec and 2011–2013 in Andalusia. The values will be calculated in 2014 US dollars.

### Comparators

The scenarios will be modelled explicitly with realistic assumptions. The scenarios to compare are QICS in Quebec and Andalusia.

The intervention is aimed at professionals involved in the CS decision-making process in hospitals. The two interventions both combine training professionals in best practices, programme implementation and technical audit of CS with feedback. The audit process makes it possible to identify inadequate CS and define corrective actions, and focuses on: 1) identifying cases; 2) data collection; 3) analysis of results and definition of actions; 4) dissemination of results and recommendations; 5) evaluating recommendations; and 6) confidentiality, legal and ethical framework.

### Variables and outcomes

Dependent variables: The primary outcome is the overall in-hospital CS rate. The secondary outcomes are as follows: a) overall rate of other obstetric interventions (intrapartum CS, planned CS, assisted vaginal delivery, pharmacological induction of labour, artificial rupture of membranes, oxytocin during labour, epidural analgesia, and episiotomy); b) vaginal birth after CS and trial of labour for women with one or more than one previous CS; and c) neonatal death.

Cost variables: Cost of the programme, including training, audits and re-certificationrecertification, delivery-related cost and complication-related cost. The cost analysis will be performed from a healthcare perspective. The data sources for the unit costs will be taken from the Patient Cost Database (Canadian Institutes of Health Research) and from Andalusia’s COAN (Analytical Accounting of the Public Health Service of Andalusia) and Official State Gazette (BOE). Discounting will not be necessary as the time horizon was less than one1 year.

Independent variables: Variables were selected *a priori* as potential risk factors for CS, at hospital and patient level, in order to take into account the differences in hospital resources and characteristics of the women who delivered in each hospital. *Hospital level:* academic status of the hospital, level of care, expenditure per capita/year, number of obstetricians/inhabitant, number of midwives/inhabitant, number of women treated per month/number of professionals, average age of professionals, average years of professional practice, sessions of continuing education/month, average age of women, percentage of children with low birth weight, percentage of women with previous CS, percentage of women with low risk, and percentage of women of foreign origin. *Patient level:* age of mother at delivery, parity, gestational age at delivery, previous caesarean delivery, any pathology during pregnancy, delivery presentation of the baby, birth weight, smoking during pregnancy, and pregnancy achieved by assisted reproductive technology.

### Data analysis

A hierarchical cluster analysis will be prerformed in each context (Quebec and Andalusia). Complete linkage will be used to identify distinct patterns of cost and CS. A dendogram will be generated to identify the number of clusters. The method will include an evaluation of the amalgamation coefficients, which provide an indication of the nature of the composition of 2two clusters at one stage of the cluster analysis being combined into 1one cluster at the next stage [21]. A sudden jump in value implies that 2two relatively dissimilar clusters have been merged; thus, the number of clusters prior to the jump is the most reasonable estimate of the number of clusters. Sudden jumps can be seen on the dendogram. Large distances between sequential vertical lines in the dendogram represent jumps in the coefficient. Amalgamation coefficients and the dendogram will be visually inspected to identify the optimal number of clusters.

As recommended in the literature, a one-way analysis of variance (ANOVA) with the Brown-Forsythe test for unequal sample sizes and unequal variance among groups will be conducted after identification of the number of clusters to confirm whether the clusters differed significantly with respect to the clustering variables.

We will use an independent- samples *t* test to evaluate whether independent variables are significantly different among clusters, taking into account the reasons for caesareans (Table 1). The chi-square test will be used to evaluate whether there was a difference among clusters in relation to the dichotomous variables. A linear model will be applied to verify the association between cost and CS, with independent variables.

**Table 1:** **Reasons for caesarean**

**Caesarean section for obstetric history**	**Fetal presentation/status**	**Anomaly labour**	**Illness or infection of the mother**	**Failure/refusal of intervention other than caesarean**	**Other indications**
*Caesarean-scarred uterus*	Death in utero	*Mechanic*	Chorioamnionitis	Failed forceps/vacuum	Caesarean section on maternal request
Failed VBAC	Premature detachment of placenta	Cephalopelvic disproportion	Eclampsia	Failed induction	
Not eligible for VBAC	Fetal distress	Multiple fibroids	Pre-eclampsia	Refusal to release	
Refusal elective VBAC	Multiple pregnancy	Ovarian cyst or fibroid previa	Genital herpes	Failure version	
Denial of VBAC during operation	Post mature	Abnormal placentation (previa, marginal)	Indication for maternal illness		
*Other History*	Prematurity (<37 weeks)	Prolapse/laterocidence cord uterine rupture	AIDS/HIV		
History of severe perineal tear	Breech presentation	*Dynamic*	Other acute maternal infections		
History of perineal fistula	Transverse	Arrest of dilatation			
History of myomectomy	Intrauterine growth retardation	Failure of progression			
History of fetal trauma	Congenital malformation				
	Premature rupture of membranes				

Separate models will be used for women at low risk/high risk and different levels of care, because previous analysis showed a clear pattern between these groups. Based on expert consensus, a woman was considered at low risk if she gave birth to a single baby in cephalic presentation, with no prior or current assisted reproductive technology, a maternal age ≥ 18 and < 40 years, a gestational age ≥ 37 and < 42 weeks, a body mass index ≥ 17 and < 30, and no previous caesarean delivery, no previous or current stillbirth, no birth defect, no *in utero* transfer in another hospital, and no other pathology or complications during the current or previous pregnancy.

We will analyse the magnitude of missing data and characteristics in order to account for missing information. We will use simple class imputation in case that missing data were considerable.

The statistical data analysis will be performed using STATA SE software.

The study will be conducted according to the established ethical principles of the Declaration of Helsinki and its subsequent revisions. Since there is no intervention, any risk to the patient is estimated. Confidentiality of information is maintained at all times. Professionals will be asked for consent to participate in focus groups, after the project and its implications have been explained to them. Data are recorded in a database that does not contain any information that could identify a particular subject.

### Facilitators and challenges for the implementation of QICS

Qualitative study. Focus group interviews were chosen for the collection of data. This method is useful for collecting data about attitudes, perceptions, and experiences [22]. The clusters and variables associated in the previous phase will alloware allowed to identifybe identified.

### Study subjects

Obstetricians and midwives. Professionals with less than 1 year of experience will be excluded. Stratified and purposive non-probability sampling following population segmentation will be performed. The participants will be recruited using a strategy of constant comparison. In order to achieve internal consistency, we will divide the population into segments based on profiles that allow the heterogeneity of participants to be taken into account: 1) Andalusia/Quebec; 1) Llevel of care (tertiary, regional);) and 3) midwives/obstetricians (Jjust in Andalusia, because midwives attend births only in 7% of Quebec hospitals) [23].

Internal homogeneity of the groups will be assessed based on age and sex. It is estimated that a minimum of 6 groups in Quebec and 12 groups in Andalusia (3 groups of each profile at each site) will be included, until achieve the saturation of data achieved.

### Dimensions

- Factors influencing delivery care at the political/management level, including local policies, leadership, organisational factors, economic incentives and the availability of equipment and staff.
- Factors influencing delivery care at hospital level, including hospital policies, culture (values, principles), the organisation of care (level of care, relationship with first and second line, management), training, quality control and risk communication mechanisms, and collaboration between services.
- Health professionals’ motivations and attitudes, legal concerns, skill levels, information and support for women, acceptance of guidelines and strategies used to implement the recommendations.
- Characteristics of mothers and their birth partner, motivations, demands and perceived needs.
- Other derived of quantitative phase.

### Data collection and analysis

Data will be collected through group interviews (focus groups) (Appendix 1). An open interview template will be used, allowing new questions to be added spontaneously during the focus groups to cover all the dimensions studied. The interviews will be recorded if participants give their consent. All recordings will be verbatim transcribed verbatim for further analysis.

We will perform the analysis proposed by Taylor [24]; the phases (adapted to our work) of which are as follows: 1) finding themes and categories by examining the data of all possible modes; 2) reduction and coding; 3) gathering and analysing all data relating to topics, ideas and interpretations related to the objectives; and 4) contrasting data considering the context in which they were collected and triangulation of results.

As a tool for encoding, we will use the software NUDIST VIVO - 8.0. To triangulate the results, we will use multiple lines of sight directed towards the same point at different times and different places. We will make a constant comparison of data from interviews of the different segments, analysed separately and checked by the researchers. External researchers will also triangulate the study.

## Discussion

The discordance between scientific evidence and clinical practice highlights the importance of studying new approaches to knowledge translation. There is a growing evidence of substantially unexplained and inappropriate variations in clinical practice patterns, concerns that further limitations in resources will reduce the possibilities of delivering high-quality healthcare, and the difficulty clinicians have in assimilating rapidly evolving scientific evidence into their practices [25]. In this context, it is essential to promote strategies for applying the evidence to medical practices, in order to improve knowledge transfer [26].

This study aims to compare the adequacy of caesareans programmes in the two regions, taking into account the two specific contexts. The objective isobjectives are to identify aspects that have been effective in changing practices and reducing the use of obstetric interventions, as well as to recognise the challenges faced by hospitals in implementing the recommendations. Since the two projects are intended to encourage and provide evidence for policy change and resource optimisation, this study would help to evaluate programmes having another context to compare with, as well as to consider the culture and organisation of care practices to support delivery-related clinical practice. The ultimate goal is to contribute to the development of policies on the appropriate use of CS.

The study uses a multidisciplinarity approach, to draw on knowledge from different disciplines: gynaecology and obstetrics, public health, health economics, and social anthropology. The objectives are to provide different perspectives on a complex problem to provide comprehensive health services [27].

Social desirability in the qualitative phase (tendency of respondents to answer questions in a manner that will be viewed favourably by others) could result in some bias in the study. In principle, this bias would be expected both in Quebec and in Andalusia. However, its influence on the results is unknown, because social desirability may be related to the specific context. Besides, comparison of quantitative results in different contexts is a complex issue, and that is why we have included a qualitative methodology prior to the quantitative analysis. This allows us to consider all the healthcare-related problems in both contexts.

Andalusia and Quebec are regions with certain similarities that invite comparisons. Both are in countries with a public health system and with similar populations (around 8 million in 2012). Knowledge management and clinical practice policies in perinatal care are being developed in both Quebec and Andalusia. The decrease in the use of non-medically necessary obstetric interventions is an objective targeted by the 2008–2018 perinatal policy of the Ministry of Health and Social Services of Quebec [28]. Andalusia has launched the Humanization of Perinatal Care in Andalusia project, which aims to use technology that ensures the safety of the mother and the newborn, and allows women and their partners to participate in the decisions of the childbirth process [29]. In 2007, the Strategy for Assistance at Normal Childbirth in the National Health System [30] was published in Spain, with a series of recommendations aimed at improving the support given to women during delivery and birth.

Variations in the performance of CS and the factors influencing this disparity have become a major area of study in recent years [31-33] This research will range factors influencing care provision in both settings in order to reduce the disparities.

There is a growing concern about the existing variation in childbirth care practice and the possible costs, in both health and economic terms, of following an interventionist model when attending to women without any obstetric risk in a highly technological environment [34,35]. The possible implementation of QICS in the healthcare system must be assessed based on its efficiency. This study will tell us which factors could be improved in each setting and in order to be implemented in hospitals.

## Appendix 1 Interviews guide

The purpose of this study is to investigate obstetricians’ perceptions of the implementation of the programme.

1. In your department, what are the factors responsible for the rise in the primary caesarean section rate?
2. What are the barriers, challenges and facilitators encountered in your practice to following or implementing the ACP at political level/management level? In terms of the following:
  - Local policies
  - Leadership
  - Organisational factors
  - Economic incentives
  - Availability of equipment and staff
3. What are the barriers, challenges and facilitators encountered in your practice to following or developing the ACP at hospital level? In terms of the following:
  - Hospital policies
  - Values, principles
  - Level of care
  - Relationship with first and second line
  - Training
  - Quality control
  - Risk communication mechanisms
  - Collaboration between services
4. What factors make the implementation or development of the programme easier or more difficult? In terms of the following:
  - Legal concerns
  - Own skills
  - Information and support for women
  - Acceptance of guidelines and strategies used to implement the recommendations
5. What are the characteristics of mothers and their birth partners that you believe to be facilitating or the follow of the programme?
  - Motivations
  - Demands
  - Needs

## Abbreviations

CS: Caesarean section
CPGs: Clinical practice guidelines
QICS: Quality improvement for CS programmes
