# Erratum to: ‘Evaluation of quality improvement for cesarean sections programmes through mixed methods’

**DOI:** 10.1186/s13012-016-0402-x

**Published:** 2016-03-16

**Authors:** Clara Bermúdez-Tamayo, Mira Johri, Francisco Jose Perez-Ramos, Gracia Maroto-Navarro, Africa Caño-Aguilar, Leticia Garcia-Mochon, Longinos Aceituno, François Audibert, Nils Chaillet

**Affiliations:** 1 Centre de recherche du CHUS, 12e Avenue Nord, Sherbrooke, QC J1H 5 N4 Canada; 2 Andalusian School of Public Health, Cuesta del Observatorio 4 s/n, 18010 Granada, Spain; 3 CIBERESP, Ciber de Epidemiologia y Salud Publica, València, Spain; 4 Division of Global Health, University of Montreal, Hospital Research Centre (CRCHUM), 900, rue Saint-Denis, H2X 0A9 Montreal, QC Canada; 5 Department of Health Administration, School of Public Health, University of Montreal, Montreal, QC Canada; 6 General Secretary of Quality iInnovation and Public Health, Consejería de Igualdad, Salud y Políticas Sociales, Junta de Andalucía, Avd. De Hytasa n° 14, 41006 Sevilla, Spain; 7 UGC Obstetrics and Gynaecology, Hospital Universitario San Cecilio, Av Doctor Oloriz, 16, 18012 Granada, Spain; 8 UGC Gynaecology, Hospital La Inmaculada, Av. Dra. Parra, S/N., 04600 Huercal-Overa, Almeria Spain; 9 Department of Obstetrics and Gynecology, University of Montreal, Montreal, QC Canada; 10 Sainte Justine Hospital, 3175 Chemin de la Côte-Sainte-Catherine, Montreal, QC H3T 1C5 Canada; 11 Department of Obstetrics and Gynaecology, Université de Sherbrooke, 12e Avenue Nord, Sherbrooke, QC J1H 5 N4 Canada

Unfortunately, the original version of this article [1] contained an error. The acknowledgements was included incorrectly. The correct acknowledgements can be found below.

The authors gratefully acknowledge the funding of this research provided by the Ministry of Health and Consumers’ Affairs - –Spain (FIS Exp. PI13/01340 and FEDER funds) and the CHIR- Quebec Training Network in Perinatal Research (QTNPR). The study funders had no role in the study design, data analysis, data collection, data interpretation or the writing of the report. The views expressed are those of the authors and not necessarily of the funding bodies.

## References

[1] Bermúdez-Tamayo C, Johri M, Perez-Ramos FJ, Maroto-Navarro G, Caño-Aguilar A, Garcia-Mochon L, Aceituno L, Audibert F, Chaillet N (2014). Evaluation of quality improvement for cesarean sections programmes through mixed methods. Implement Sci.

